# An Active Model of Research Translation for the General Public: Content Analysis of a YouTube-Based Health Podcast

**DOI:** 10.2196/46611

**Published:** 2023-12-05

**Authors:** Maka Tsulukidze, Stuart W Grande, John A Naslund

**Affiliations:** 1 Department of Health Sciences Marieb College of Health & Human Services Florida Gulf Coast University Fort Myers, FL United States; 2 Division of Health Policy and Management School of Public Health University of Minnesota Minnesota, MN United States; 3 Department of Global Health and Social Medicine Harvard Medical School Boston, MA United States

**Keywords:** evidence translation, user engagement, consumer education, online health information, disseminating science, health education

## Abstract

**Background:**

Online health information seeking is changing the way people engage with health care and the health system. Recent changes in practices related to seeking, accessing, and disseminating scientific research, and in particular health information, have enabled a high level of user engagement.

**Objective:**

This study aims to examine an innovative model of research translation, The Huberman Lab Podcast (HLP), developed by Andrew Huberman, Professor of Neurobiology and Ophthalmology at the Stanford School of Medicine. The HLP leverages social media to deliver health information translated into specific, actionable practices and health strategies directly to the general public. This research characterizes the HLP as an Active Model of Research Translation and assesses its potential as a framework for replicability and wider adoption.

**Methods:**

We applied conventional content analysis of the YouTube transcript data and directed content analysis of viewers’ YouTube comments to 23 HLP episodes released from January to October 2021, reflecting the time of data analysis. We selected 7 episodes and a welcome video, to describe and identify key characteristics of the HLP model. We analyzed viewer comments for 18 episodes to determine whether viewers found the HLP content valuable, accessible, and easy to implement.

**Results:**

The key HLP features are direct-to-the-consumer, zero-cost, bilingual, and actionable content. We identified 3 main organizing categories and 10 subcategories as the key elements of the HLP: (1) Why: Educate and Empower and Bring Zero Cost to Consumer Information to the General Public; (2) What: Tools and Protocols; Underlying Mechanisms; and Grounded in Science; (3) How: Linear and Iterative Knowledge Building Process; Lecture-Style Sessions; Interactive and Consumer Informed; Easily Accessible; and Building the Community. Analysis of viewers’ comments found strong consumer support for the key HLP model elements.

**Conclusions:**

This Active Model of Research Translation offers a way to synthesize scientific evidence and deliver it directly to end users in the form of actionable tools and education. Timely evidence translation using effective consumer engagement and education techniques appears to improve access and confidence related to health information use and reduces challenges to understanding and applying health information received from health providers. Framing complex content in an approachable manner, engaging the target audience, encouraging participation, and ensuring open access to the content meet current recommendations on innovative practices for leveraging social media or other digital platforms for disseminating science and research findings to the general public, and are likely key contributors to HLP impact and potential for success. The model offers a replicable framework for translating and disseminating scientific evidence. Similar active models of research translation can have implications for accessing health information and implementing health strategies for improved outcomes. Areas for further investigation are specific and measurable impacts on health, usability, and relevance of the model for reaching marginalized and high-risk populations.

## Introduction

The past decade has witnessed significant transformations in the way scientific research and, in particular, health information are sought, accessed, and shared. New ideas such as Medicine 2.0, PHR 2.0 (Personal Health Records integrated with social networking) [1], and Science 2.0 have emerged to characterize this novel trend that blends the widespread use of Web 2.0 technologies with the creation and distribution of scientific knowledge. These new trends share the same principles of participation, engagement, and collaboration in the continuum of knowledge production and dissemination [2].

Digital technologies have transformed attitudes and culture within the field of health care [2]. These interactive technologies have facilitated a significantly higher level of user engagement than ever before in history [3]. According to a Pew Research survey, in 2013, nearly 35% of US adults searched for health information online, either for their own health or that of someone they knew [4]. A recent study, which analyzed data from the Health Information National Trends Survey spanning from 2008 to 2017 [5], revealed that over 81.5% of adults in the United States had, at some point, sought health information online. Furthermore, 68.9% of adults (95% CI 67.8%-70.1%) reported that they initially turned to the internet when searching for health information. In the same survey study, the proportion of adults who indicated that they accessed health information on the internet without experiencing any frustration rose from approximately 31% in 2008 to nearly 43% in 2017 [5]. The growing trend of seeking health care and health information outside of traditional health care settings [6] signifies a new approach to harnessing web technologies for accessing and obtaining high-quality, trustworthy health information. This swift expansion in the dissemination of health information through online platforms includes patient education portals (like Interactive Patient Education [7]) and initiatives focused on patient engagement (such as the Patient Empowerment Network [8]), as well as health education blogs and podcasts covering a wide range of health-related topics.

While the accessibility and availability of health information on the internet are likely to benefit end users, it is important to acknowledge several key challenges: (1) fragmented information, (2) limited link between online information and tangible health improvements [9], and (3) credibility of online source material [10].

Additional examples of practical health-related knowledge translation are the Centers for Disease Control and Prevention Rapid Synthesis Translation Process [11], Best Practices Guide for Cardiovascular Disease Prevention Programs [12], and Best Practices Framework [13]. However, these approaches see limited adoption in practice [12] and require structured interventions delivered by health professionals. Numerous strategies have been used in specifically supporting the communication of science and health information through digital platforms, as well as recent guidelines aimed at facilitating the dissemination of research findings to the public [14]. A recent review revealed that the various communication platforms can be broadly categorized into social media platforms (e.g., Twitter/X [X Corp.] or Instagram [Meta Platforms]), content-sharing platforms (such as YouTube [Google LLC]), digital research communities (such as ResearchGate [ResearchGate GmbH]), personal blogs and websites, and social news aggregation platforms and forums [15]. Nevertheless, there has been a scarcity of studies that specifically delve into how various strategies are used on these diverse platforms to effectively convey science and health information. Furthermore, to propel the evolution of Research Translation 2.0, a more focused approach is essential for translating evidence for end users.

This research aims to examine an innovative model of research translation, The Huberman Lab Podcast (HLP), developed and delivered by Andrew Huberman, Professor of Neurobiology and Ophthalmology at Stanford School of Medicine. The HLP leverages social media to deliver health information translated into specific, actionable practices and health strategies directly to consumers.

**Figure 1:**
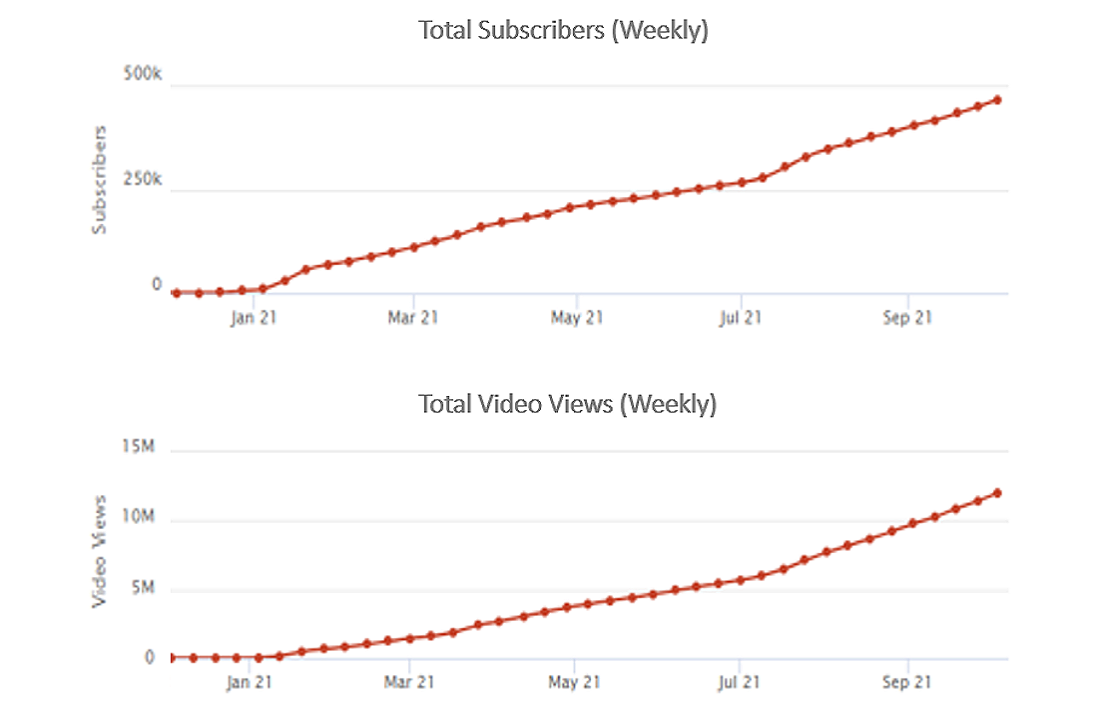
Total subscribers and video views (weekly). Data from [16,17].

**Figure 2:**
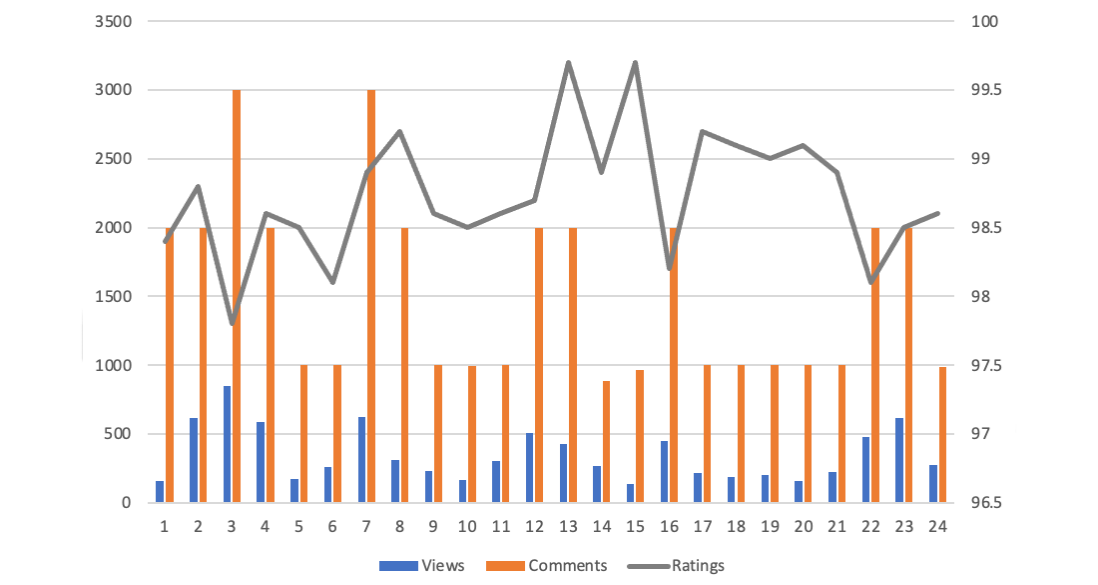
Views, comments & ratings for 23 HLP episodes and welcome video. HLP: Huberman Lab Podcast.

A rapid assessment of the podcast found that a vast majority of YouTube commenters reported consistent engagement with the podcast content, self-reported improvements in their health and well-being, and expressed continued interest in watching future episodes. The subscriber numbers grew rapidly soon after the HLP was launched in January 2021, with weekly views showing a steady growth (Figure 1) [16,17]. Viewers felt compelled to comment on each episode and comments ranged from 500 to 3000 per episode. Similarly, ratings (percentage metric derived from “likes” and “dislikes”) consistently stayed between 97% and 99% (Figure 2).

Key features of the HLP credibility, systematic and methodical synthesis of scientific evidence, nonfragmented information delivery, active and direct end user engagement via social platforms, and their interest in specific, actionable protocols stood out to us as a compelling approach. We sought to examine the HLP model as a potential framework for research translation. The purpose of this work, therefore, is to characterize the HLP as a model of research translation and assess its potential for wider adoption and application in the practice of science translation and dissemination.

## Methods

### Study Design and Source

This qualitative study examined comment and transcript data from the HLP on YouTube, an open-source media platform where the public can view content and post comments. Transcripts were extracted from selected episodes using YouTube’s transcript-generating feature and saved as PDF documents. Comment data were extracted for each HLP episode and saved as PDF documents. All data were extracted in June 2021.

### Study Data

The data sets analyzed during this study are available publicly on the HLP YouTube channel [18]. Transcripts and viewer comments for each specific episode included in our analysis can be obtained by accessing the selected episode. For example, transcripts and comments for the Welcome video can be accessed at a corresponding URL under the HLP YouTube channel [19]. Time-stamped PDF documents with transcripts and viewer comments saved by the researchers during the data collection and data analysis phases will be provided upon request.

After a data familiarization stage that included the initial coding of transcripts, we made the following decisions on data inclusion: out of a total of 23 episodes released by the time of data analysis, we selected transcripts of 7 episodes (episodes 1-6 and 23) and a Welcome video, as they described Huberman’s approach, philosophy of research translation, and foundational principles for the HLP. Therefore, we decided that analyzing these episodes would allow us to describe and characterize the HLP model of research translation, establish, and identify key characteristics of his model as introduced in these early episodes. Episode 23 was added to the analysis as we noted that it reflected a change in organizing the episodes and corresponding resources. Specifically, all episodes were organized on the Huberman Lab website [20] with links to protocols and other resources. No additional episodes offered any new insights regarding the HLP model.

Of the 23 time-stamped PDF documents with viewer comments, we analyzed 18 (1-9, 12-19, and 23), which enabled us to determine whether viewers found the information shared in the HLP valuable and helpful and if they found it accessible and easy to implement in everyday practice. Twenty-two episodes, released by the time of data collection, were initially divided between 2 researchers for analysis (SWG: 1-11 and MT: 12-22). All viewers’ comments for the selected 22 videos were saved as PDF files. However, after independently identifying initial coding categories, the researchers (MT and SWG) determined that data saturation was achieved (episodes 1-9 MT and 12-19 SWG). Therefore, the final subset for the data analysis comprised 18 episodes, as indicated above. Episode 23 was added to the analytical subset after Huberman announced that starting from this episode, all podcast episodes would be organized on a dedicated web page [20] with linked protocols and other related resources to each episode.

### Content Analysis

We applied 2 distinct analytical approaches to examine the HLP as a potential framework for translating scientific evidence into actionable protocols and evaluate its potential for replicability and wider adoption. The first centered on using conventional content analysis [21] of the podcast transcripts to identify the key elements of the research translation model and define its main characteristics. The second applied directed content analysis to viewers’ YouTube comments for 23 episodes to find evidence for the value or applicability of the HLP content. The selected episodes aligned with Huberman’s stated progression of content as part of the podcast design.

We analyzed the podcast transcripts using a generalized coding technique that included labeling text. Two researchers (MT and SWG) independently identified key concepts of the model as initial coding categories (eg, descriptions of how content was framed, delivered, organized, and summarized by analyzing a set of HLP episode transcripts). A working model of the podcast was derived through an iterative process of coding selected transcript data, organizing codes, memo writing (summaries), and weekly discussions. This process identified key operating principles governing the creation and implementation of the HLP. Subsequent weekly meetings were conducted to keep the code list up to date, to compare codes for consistency and clarity, and to share insights when necessary regarding emerging codes or concepts extracted from the data.

Directed content analysis of YouTube comments enabled the extraction of concepts or language describing viewers’ assessment of the content, their personal experience related to the HLP, and their views on the value of the podcast. Coding of comments focused on identifying narrative signals in the text that represented how users reacted to the podcast content, how viewers interpreted the content, why they chose to listen, and ways the content resonated or confused them. Coding continued until patterns or characteristics of the data were identified and no new information or insights emerged. Authors define this step as data saturation, at which point no additional analysis would be necessary.

The analysis process was inductive wherein we let the categories emerge on their own. During the analysis, the coding of data was subject to iteration, testing intercoder agreement, and revising the coding scheme. Whenever the intercoder agreement did not reach an acceptable level, determined through discussion and having the coders review each other’s codes, or new insights emerged from the data analysis, the coding categories were revised accordingly. The coding categories were designed to be mutually exclusive (distinct from each other) and exhaustive.

As a measure of engaging consumers in the HLP content creation and delivery, we separately analyzed the replies posted by Huberman answering viewers’ questions, offering comments or other insights. In addition, we collected and analyzed YouTube web metrics (eg, views per episode, comments, likes) to further assess consumer engagement and interest.

### Ethical Considerations

The University of Minnesota Institutional Review Board reviewed the protocol and found this research did not require additional review as it did not meet the criteria for human subjects–based research.

## Results

### Overview

Launched in January 2021, the HLP quickly acquired YouTube subscribers and views with a steady weekly growth (Figure 1). Viewer comments range from 500 to 3000 per episode and ratings (percentage metric derived from “likes” and “dislikes”) consistently stay between 97% and 99% (Figure 2). Illustrative quotes are included verbatim.

### HLP Model Characteristics

**Figure 3:**
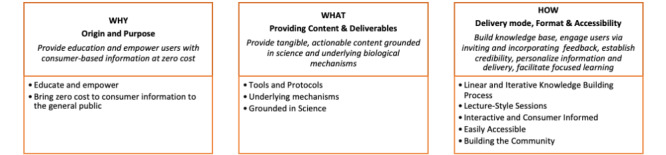
Elements of the HLP model. HLP: Huberman Lab Podcast.

We arranged initial findings drawn from conventional content analysis of HLP episode transcripts into 3 main organizing categories: (1) Why—Origin and Purpose; (2) What—Content and Deliverables; and (3) How—Delivery Mode, Format, and Accessibility (Figure 3). These categories were generated through discussion during the process of data analysis and are presented below. Each consists of corresponding codes with examples and descriptive evidence. We reference the episode and PDF page numbers for all quotes.

### WHY—Origin and Purpose of the Podcast

#### Overview

This category provides guidance on why HLP was created and the purpose it serves. The viewers are guided on what to expect and how to apply the information.

#### Educate and Empower; Bring Zero Cost to Consumer Information to the General Public

The purpose of the HLP was defined by Huberman in an early episode where he frames it as an obligation to serve by providing “...zero cost to consumer information about science and science related tools to the general public” [introduction statement for all episodes].

Huberman views translating research as a form of educating and “arming” people “with information” (E3, P96) that empowers them to be in control of their health and well-being. In addition to educating, there is a strong presence of empowering language both in the way Huberman talks to viewers and narrates the podcast.

### WHAT—Providing Content and Deliverables

#### Overview

The primary component of the podcast is a narrative content framed around “science-based tools” that can be applied in daily life. Simultaneously, Huberman explains the complex “biological mechanisms” underlying these tools and provides detailed scientific evidence to support any data, findings, reports, or suggestions presented during the podcast.

We identified 3 subcategories: Tools and Protocols, Underlying Mechanisms, and Grounded in Science

#### Tools and Protocols

Huberman defines tools and protocols as both technical and informational instruments to help listeners achieve their health and lifestyle goals. These were characterized early in the podcast in the following way:

I just briefly wanna touch on what I mean by tools. Some come in the form of behavioral tools, so things that you would want to do to get a particular result Other tools are about not doing certain things Still other tools relate to things like nutrition or supplementation or prescription drugs or brain-machine interface, devices that you use to monitor your nervous system and biology, or to change your nervous system and biology. We're gonna cover all of that as well as their various advantages and disadvantages.

Welcome, P3

Although the tools and protocols serve as guides and methods for utilizing some of the research and findings shared on the podcast, Huberman urges viewers to be safe and cautious in their application:

Your healthcare, your wellbeing is your responsibility. So anytime we talk about tools please filter it through that responsibility. Talk to a healthcare professional if you're going to explore any new tools or practices and be smart in your pursuit of these new tools.

E1, P5

Starting from episode 23, all podcast episodes were organized on a dedicated web page [20] with linked protocols and other related resources to each episode. A protocol for episode 23 is a printable PDF file summarizing the 4 types of endurance training covered in the episode, with specific target approaches and examples for each.

#### Underlying Mechanisms

A distinguishing feature of the HLP is the use of unabridged scientific mechanisms to explain the technical characteristics of key physiological and neurological processes. Huberman provides an overview of anatomical and physiological concepts “to equip you with a language so that we’re all developing a kind of common base set of information going forward.” [E1, P43]

In some instances, descriptions are highly technical, while in others, like this example from the first episode, he details “the parts list of the nervous system” as

The bits and pieces that together make up everything about your experience of life, from what you think about to what you feel, what you imagine, and what you accomplish from the day you're born until the day you die.

E1, P3

Across all episodes, descriptions like this serve as the principal component for a given topic. These often follow episode introductions and provide a clear biological and physiological picture of the underlying science behind daily human function and experience.

[...] understanding mechanism affords you more flexibility. You understand how to control the machine that is your biological system, your nervous system.

E4, P4

#### Grounded in Science

As part of how Huberman delivers content through the podcast, evidence is foregrounded to eliminate any concerns relative to the veracity of the science. He does this by making a point to separate science from myths and grounding his discussion in the available evidence. He emphasizes that he is going to “ground our discussion of tools in peer-reviewed studies.”

The word neuroplasticity means so many things to so many different people that I thought it would be important to put...organizational logic around what it is and how it happens, because if you were to Google the word neuroplasticity, you would find hundreds of thousands of references, scientific references as well as a lot of falsehoods about what neuroplasticity is and how to access it. As I mentioned before, we're going to talk about the science of it and we're going to talk about the tools that allow you to engage this incredible feature of your nervous system.

E6, P3

### HOW—Delivery Mode, Format, and Accessibility

#### Overview

Several strategies are used to organize the HLP content. In some instances, Huberman is explicit about how the information is presented while in other instances the data are characterized by certain patterns and features.

We identified the following subcategories: Linear and Iterative Knowledge Building Process, Lecture-Style Sessions, Interactive and Consumer Informed, Easily Accessible, and Building the Community.

#### Linear and Iterative Knowledge Building Process

The podcast is organized in a linear, iterative pattern, where information is presented by topic each month. Topics are revisited to highlight areas where more explanation is needed, or the interconnectedness of the topics is emphasized.

We will revisit a lot of these themes going forward. So if all of that didn't sink in in one pass, please don't worry. We will come back to these themes over and over again.

E1, P43

Each topic is structured into 4-5 episodes, which enables the creation of a knowledge base for that specific topic and fosters a better understanding of the subsequent topics.

On the Huberman Lab Podcast, we're going to dive deep into individual topics for an entire month at a time. So for instance, we might take an entire month and go really deep into the topic of motivation and focus and talk all about the science underlying motivation and focus, what's known, what's not known, and then discuss the various tools as well as some of the barriers to motivation and focus that exist out there as a way for you to really understand how these processes work within you and within the people that you know

Welcome, P1

#### Lecture-Style Sessions

For the 23 episodes analyzed, information was delivered uninterrupted in a lecture-style manner. Based on previous narrative data, the mode of delivery was determined a priori.

Sometimes, it’s going to be just me talking to you, other times, it's going to be a guest ...with me.

Welcome, P2

In terms of organizing the content, it follows a specific structure, where general context and reviews of previously covered (applicable) concepts lay the foundation for additional discussion.

I promise you're going to understand a lot more about how you work and how to apply that knowledge. There's going to be a little bit of story. There's going to be a lot of discussion about the people who made these particular discoveries. There'll be a little bit of technical language. But at the end you're going to have in hand what will be the equivalent of an entire semester of learning about the nervous system and how you work

E1, P4

#### Interactive and Consumer Informed

The design of the podcast supports active participatory interaction between Huberman and the audience. An example of this principle of engagement comes from the feature he introduced as “office hours,” where he asks his viewers to pose questions and offers to respond.

It's sure to be a very rich discussion back and forth where I'm answering your questions and providing tools. And I’m certain you’re also going to learn a lot of information about neuroscience

E1, P44

An invitation to provide comments and feedback intends “to actively shape the direction of the podcast” [Welcome, P3]. Viewers were reassured that all comments would be reviewed, and feedback incorporated into the content. Huberman answers questions, provides clarifications, or corrections as appropriate.

There's another thing that's unusual about this podcast, which is that you, if you choose, can be a very active participant by placing your suggestions about which topics to cover and which information you would like to learn more about in the comment section. We are going to pay careful attention to those comments and the number of likes that those comments receive. So the comment section is a place for you to give us constructive criticism, give us praise if you like, but most importantly it's going to be a place for you to make suggestions about what you would like to hear more about, and we will respond to that. please upvote or vote for the things that you want to hear more about.

Welcome, P2

One of the notable features of the HLP model is the level of personal engagement. Huberman replies to an average of around 30 viewer comments for each episode.

These direct replies to comments reflect a variety of emotional or sentimental characteristics. They tend to be short, 1-2 sentences in length, and vary with the level of personalization. Replies often use friendly wording that appears appreciative, empathetic, inquisitive, and often validates the viewer’s comment or acknowledges a viewer’s hardship. Outside of the style or emotion of the replies, their content includes 3 main response types, namely, informational, reflective, and explanatory.

Informational responses tend to provide basic information to the comment. One helpful example drawn from episode 15 is a reply to a question about the fee for content:

We have no intention of placing this behind a pay wall. I am able to support a talented and dedicated production staff, translators, etc. thanks to sponsors mainly. But even for those that don’t explore those, I want people to know that this channel is for you and those who want to learn. Free education is the foundation here.

Another type of reply was a reflective response. These responses feature opinions often supported by evidence or a reference to peer-reviewed literature and detail a thought or process that supports the overall approach of the podcast itself. A good example is from a reply to a question about the quality of evidence shared:

I tend to only emphasize work that has a strong center of mass meaning several studies from quality groups etc. Occasionally I will refer to work that was based on one single study and has not been replicated but in general I lean towards things that have been replicated.

Explanatory responses may be described as answering a question in the comments on a shared protocol, a nuance in a referenced study, or a follow-up clarification. One example is a clarifying reply about how WimHof breath holds produce dopamine and serotonin:

I’m not aware that extended breath holds have that effect. Thus far no science to support that claim. It may be the case but I’m personally not aware of any evidence for it. Epinephrine from the cyclic deep breathing, yes. Dopamine and serotonin -seems very unlikely. Thank you for your question and feedback.

#### Easily Accessible (Free, Time-Stamped, Bilingual, Duration Built Around Ultradian Rhythm)

Unrestricted access is provided for the entire podcast content. Episodes are time-stamped for ease of access and viewing. Spanish subtitles are provided by a professional Spanish translation service.

The episodes were initially structured to last 90 minutes, to match an ultradian cycle for better focus and learning.

The most important ultradian rhythm for sake of this discussion is the 90 minute rhythm.... And it turns out that we are optimized for focus and attention within these 90 minute cycles

E1, P40

However, later episodes became of longer duration, so Huberman encouraged the use of time stamps to access information as convenient.

#### Building the Community

A notable feature of Huberman’s engagement and interaction with viewers is his active attempts to bring the viewers together to build a community of learners.

...recommend it to a friend, the community that we're creating here around these topics...is best supported by your involvement and your questions.

E2, P99

To further engage consumers, Huberman also integrated his dog, Costello. While it may appear to scientifically hold little value, it created a sense of connection and closeness.

There's one other thing that's unusual about this podcast, which is that I have a very large 90-pound bulldog mastiff named Costello. He is an active participant in the Huberman Lab, he's a steady member of the podcast, so you can look forward to more Costello appearances in the future.

Welcome, P3

### Consumer Interest and Engagement

Viewers’ comments for the 18 episodes included in our analysis ranged from 500 to over 3000 per episode (at the time of analysis). As we applied directed content analysis to these comments to determine if core elements of the HLP were recognized and accepted by the viewers, we found that several elements were particularly strongly supported. Specifically, *Educate and Empower, Tools and Protocols, Consumer Informed Content,* and *Easily Accessible* were the most represented in terms of the number of comments.

Viewers describe the HLP as transformative and empowering, “one of the most impactful podcast [sic] ever made” [E12, P72], feel “better equipped” [E13, P36] to “have more control over my life” [E13, P40], and often describe the content as “gold,” “gold mine,” “wealth of knowledge,” “gold standard in evidence-based content for improving life outcomes” [E19, P25]. They note improvements in their health and well-being after applying “a plethora of actionable tools” [E17, P15] convey an enormous sense of gratitude and offer specific examples of personal and professional impact. These examples range from improvement in sleep, motivation, focus, and general well-being (eg, becoming “much healthier so quickly” [E12, P24]) to finding relief for specific diseases and disorders (eg, bipolar disorder [E12, P49; E18, P71], posttraumatic epileptic seizures [E15, P95], and drug addiction [E17, P35]).

Comments point out that information is useful for filling the gaps in clinical care, “the information is helping me when dozens of doctors have been unable to” [E12, P90]. Others note how information “fills a HUGE deficit in my search for info on this topic ❤” [E12, P40] and many view “Dr. Huberman is a doctor I trust” [E15, P77]. Those who lack access to health care also note how important this knowledge is as now they “can take care of my health in an efficient way” [E18, P56].

For many, the interactive format offers an opportunity to have a voice in informing the content as well as to clarify their questions. Viewers actively post questions and request coverage of specific topics. Many thank Huberman “for reading the comments and incorporating the themes into your new videos” [E13, P56] as he “consistently answer the questions that I’ve always wondered about but did not know who to trust” [E18, P25].

Viewers also find the HLP easily accessible in many important aspects: (1) free access—some express fear, “anxiety attacks that it will not be free forever ☺” [E15, P40] and download “all of your videos to have it forever” [E18, P40]; (2) bilingual with Spanish subtitles—many ask for subtitles available in more languages including French, Japanese, Check, Ukrainian, Russian, and Italian and offer help with translation; (3) accessible for all age groups from children of 7 and 10 years old to the “granpa” who “is loving it!” [E18, P44], and a “member of a senior community...who shares ‘much of this new knowledge’” [E15, P86]; (4) clear and understandable as “complex” information is shared “in a way some of us non-science folk can digest” [E16, P37] while keeping it engaging and interesting for “academia-nuts” [E13, P33]; (5) “a simple well thought out format” [E12, P58], “a clear, organized, concise, INTERESTING” [E17, P15] delivery of information and circling “back to previous topics to draw the important connections and help us deepen our understanding!” [E10, P20]. They also recognize that Huberman is “thorough...who leaves very little room for confusion” [E17, P17].

Many viewers express the sentiment that “this is the future of education” [E12, P52] and “wish all important knowledge was this plentiful, accessible, and free. This should be a standard template for all academic fields” [E17, P14].

**Figure 4:**
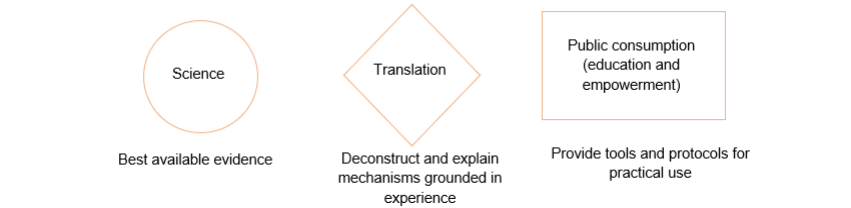
HLP model as a framework. HLP: Huberman Lab Podcast.

Figure 4 describes the HLP model as a framework.

In Table 1, we map supporting evidence to each HLP element and provide illustrative quotations.

**Table 1:** Mapping supporting evidence from YouTube comments to the HLP^a^ model elements.

HLP model element categories and subcategories		Summary of supporting evidence from YouTube comments	Representative quote(s)
**Why—** **Origin and Purpose**			
	Educate and Empower; Bring Zero Cost to Consumer Information to the General Public	Podcast described as “life-changing”, “enlightening and empowering” [E13, P65], and “feel smarter and more in control over my health” [E19, P15].	You should know that watching you mindfully is transformative. This is honest. [E23, P18] Not to sound dramatic, but stumbling upon your work has been powerfully transformative. In the short time I've been watching this podcast I've dramatically improved my sleep, revved up my ability to focus, tamed my stress levels and I'm no longer at the mercy of unwanted fatigue. [E16, P13] At the end of the day...I hope that you know you make a big difference in my life. Thank you. [E13, P29]
		Huberman’s expression “scientists of yourselves” resonates; knowledge of underlying mechanisms enables them identify the reasons behind their challenges and address them accordingly.	quote of the day for me “...as scientists of yourselves...” All of these lectures have been instrumental in helping me cope with a rare autoimmune disorder. Feeling better every Monday. [E17, P12] Thank you Professor Huberman, for helping me become a scientist of my own body and mind. You are a blessing! [E12, P67]
		Service to the general public appreciated	Key intro info... “... part of my desire and effort to bring zero cost to consumer information about science and science related tools.” [E14, P31] I never leave a comment and i’m on YouTube everyday absorbing information, i listen all your podcasts its amazing how you explain everything don’t stop please,keep educate us!!! Thank you Doc!!! [E12, P66] This is a real service you are doing. Your work has helped me to understand the neuroscience behind my behaviors, and I'm using that info in my business and my recovery. Thank you. [E12, P72]
**What—** **Content and Deliverables**			
	Tools and Protocols	Each episode provides actionable, practical tools and protocols that can be implemented in everyday life.	People could, if they wanted to, build their own personal development plan based on what they learn from this podcast! I will be eager to see how much I've progressed after integrating what I've learned. [E16, P40] Almost every video has somehow applied to making myself into a happier, healthier, or smarter person. Thanks Professor Huberman, you have no idea how awesome it is that you take the time to put these out! [E15, P14]
		“Easily accessible tools that anyone can adapt” [E16, P30] are helpful “to be more strong and resilient” [E18, P28]	Ihave bipolar type 1 and have been sober from drinking for 2 years, you have given me so many tools to help me in my journey. [E12, P49] I never thought I would ever find neuroscience so interesting or be smart enough to understand it. These podcasts and tools have literally changed my life...the only way I can repay you is to share them with people, Thankyou Dr Huberman.. [E13, P26]
	Underlying Mechanisms	Interest in the explanation of underlying mechanisms “going in depth with ‘whys and hows’” of it. [E19, P28]	This is life changing stuff for me. I've had dozens of doctors tell me to do xyz, but it never sticks. Turns out I NEED to know the underlying mechanisms to make change. [E18, P44]
		The belief that they “need to learn the mechanisms to put things into practice [...] and leverage that.” [E18, P78]	I’m so incredibly thankful to have this man explaining to me how the body works, why it works like that, and ways to control it. [E17, P13]
	Grounded in Science	The podcast is seen as “scientifically informative (with all those great practical tips)” [E12, P105] with information grounded in scientific evidence.	You're not getting opinions here, you are getting science presented in a digestible and relatable format.. [E17, P70]
**How** **—Delivery Mode, Format, and Accessibility**			
	Linear and Iterative Knowledge Building Process	Information provided “in such a simple well thought out format” [E12, P58] “appreciate that you circle back to previous topics to draw the important connections and help us deepen our understanding!” [E10, P20]	I also really appreciate how this episode puts together and builds on information from prior ones [E18, P28]
	Lecture-Style Sessions	The manner in which the content is delivered is seen as clear, concise, and organized, “presenting science in such a clear, fascinating and capturing manner, it's really awesome!” [E13, P48]	One thing I've really appreciated is HOW you present information in such a consumable way. It's obvious that you are a skilled teacher [E4, P41]
	Interactive and Consumer Informed	Follow-up questions seeking clarification, offering insights, and requests for coverage of specific topics, for example, traumatic brain injury, posttraumatic stress disorder, attention-deficit/hyperactivity disorder, autism, dyslexia, hormonal balance, general wellness, and health. Interactive format appreciated, grateful for “reading the comments and incorporating the themes into your new videos” [E13, P56]	Really answering and acknowledging your listeners' questions and comments (just like you said you would) Your style cannot be matched or duplicated, Sir. [E14, P51]
	Easily Accessible (Free, Time-Stamped, Bilingual)	Free access, bilingual, accessible for all age groups, clear, and easily understandable	Unbelievable that you do these lectures out of your own time and for free. I find it insane that I can access this sort of information and be as invested as I am towards them. Hats off to you from across the sea [E18, P17] Thank you sooosoo much for the Spanish caption. I can now share them with my mom since I talk to her about some of the things you mention on here. I can now send them to her. [E12, P35]
	Building the Community	The sense of community is very strong and is expressed in various ways, including extending support to each other, providing references to useful sections of a specific episode, clarifying questions when they can, and using a shared language, which often references what Andrew Huberman discussed or joked about. A community of learners share notes, create an app called Costello (in reference to Andrew Huberman’s dog), which combines information from all episodes, makes it searchable, and creates a discord group where all interested podcast viewers can participate.	who's here every week?? 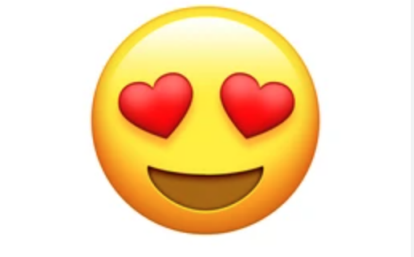 Hey Fam❤ [E16, P13] [note: this comment has generated 22 replies with all commenters noting that they are happy viewers of “ Dr Huberman's Youtube lab”]
		Positive comments, gratitude, and respectful feedback	This is probably the most positive comment section on all youtube xD Huberman, you do magic [E17, P18]
		Supporting the podcast so that others can benefit from it	“Share it with your friends, family and colleagues”. I tell strangers about this. [E13, P55] THANK YOU for creating a Patreon account! It means a lot to me personally to support your work. [E12, P121] I literally put my business on hold to study this stuff full time bro, 15 years in the game suffering from bipolar, looking for answers [..] Bro a big part of me had given up, [...] you gave me a new lease, [...] I will devote the rest of my life to spreading this stuff to the bipolar community, stay cool brother! [E18, P71]

^a^HLP: Huberman Lab Podcast.

## Discussion

### Principal Findings

We analyzed an innovative Active Model of Research Translation and identified its core elements. Our findings show that this model can facilitate timely evidence translation using direct and effective consumer engagement and education, and it provides actionable protocols offering the potential to improve the health and well-being of viewers. The key features of the model are content that is direct to the consumer, zero cost, bilingual (Spanish and English at the time of analysis), grounded in real-life, and clear. These appear to improve access and confidence related to the use of health information and may potentially reduce known challenges for patients concerned with understanding and applying health information received from their physicians or other health providers.

Comparatively, other models of health communication that predate social media may be framed as either promoting protection behavior or avoiding risk behavior [22]. In an era where social media and the internet are driving consumer behavior, older models of health communication may require some new thinking to match. This view of communication applies to models for health promotion and behavior change, whose efficacy and applications have been well supported in the literature [23]. Borrowing from Greenhalgh and Wieringa’s [24] framing of “knowledge translation” in medicine as a means of addressing the “know-do gap,” we might consider Huberman’s model as one that aligns well, serving to effectively link practical wisdom with evidence-based practice. The Active Model of Research Translation also appears consistent with this new format of knowledge translation by avoiding the language of behavior change or behavioral modification and presenting “knowledge” through a mode (such as YouTube) that matches user demand with accessibility. The HLP seems to achieve this by promoting best practices through explicit statements and references to systematic review evidence, leaving viewers to assess basic protocols on their evidence-based merit, and not relying solely on the guidance of the speaker. Basing any translational mode on systematic review evidence is viewed as the best practice by implementation science.

Looking at other models of either knowledge translation or research translation in medicine, we see a much broader array of approaches particularly around the uptake of best practices for clinicians’ use rather than for patients [25]. According to a recent structured review, there are only a few knowledge translation frameworks that guide best practice, which suggests that the HLP, among other novel modes of integrating research or knowledge into practical consumer tools, is breaking ground [26]. Given that social media and other internet-based knowledge translation models have been used broadly for educating or communicating updated research for clinical care, there are a few reviews or characterized models that frame this type of translation for direct patient use. Additionally, there have been guidelines or recommendations published on innovative practices for leveraging social media or other digital platforms for disseminating science and research findings to the general public [14]. These recommendations have centered around key principles in framing complex content in a useful and approachable manner, engaging the target audience, encouraging participation, and ensuring open access to the content [14], all of which are also reflected in the HLP and are likely key contributors to its impact and potential for success. What is also unique about the HLP is the co-production of content, which has been described by Dal Mas and colleagues [27] as a novel means of leveraging the benefits of knowledge translation in partnership with end users. Such a perspective validates the consumer-driven portion of the HLP as a means of co-producing meaning between experts and end users through the medium of YouTube interactions.

As an Active Model of Research Translation, the HLP is designed as an interactive and user-informed digital product. The impact of the model, as characterized by user acceptance and interest, stands on 2 main pillars—clear educational content and actionable, specific, science-based tools and protocols. Protocols outline 3 specific areas, namely, what (eg, sleep, nutrition, and exercise), when (eg, viewing sunlight in the morning), and how (eg, specific strategies, supplements, and dosage). Recognizing these are not medical advice; instead, viewers are reminded to seek clinical care as needed. This Active Model of Research Translation, which includes real-time public feedback to inform and update dissemination features, fits well within the context of patient-informed research as described by integrated knowledge translation. As Banner and colleagues [28] point out, the meaningful engagement of the public [read patients] in the processes of collecting and disseminating health information is directly connected to the public’s willingness to accept and apply medical knowledge and evidence to their daily lives.

### Limitations of the Study

While the model offers a replicable strategy for translating evidence into actionable tools and provides a clear framework for their dissemination directly to end users, we note several considerations about this model and acknowledge limitations in our ability to assess its success. The model has been launched and utilized for less than a year (since January 2021). Therefore, our analysis must be considered preliminary given that long-term impact and metrics for the videos are not available. The analysis could not determine characteristics of podcast viewers beyond self-report, potentially limiting generalizability to lay audiences. It is also not possible in our study to determine viewers’ demographics, meaning that we do not know whether the HLP is reaching underrepresented racial and ethnic minority groups, older viewers, or lower income or lower literacy viewers. Further, our analysis included a small subset of data from a very early stage of the HLP, which continues to grow as new episodes are released weekly. During our analysis, a few changes were implemented to make information more easily accessible and digestible. While some changes may not be captured in our data, our analysis has enabled us to identify the key principles that are foundational to this model and can be adopted in other areas of evidence translation.

Assessing evidence delivered in the podcast was outside the scope of this research. Notably, Huberman mentions consulting subject matter experts in episode preparation. Further, the open-source platform invites a certain level of scrutiny from viewers and outside experts. If this model is adopted and replicated, our recommendation would be to complete evidence translation in a peer-reviewed, team-based format.

Our analysis showed that large numbers of users self-reported positive impacts from the content of the podcast episodes, such as improvements in mental health, overall well-being, and specific disease areas. However, it is important to note that these reflect self-reported feedback in the comments under each video, and that additional research should examine specific and measurable impacts on health. Given the exploratory nature of this study and the use of publicly available content shared on social media, it is not possible to link the use of the HLP to the objective measurement of benefits. It is also not possible to determine the demographics of the viewers of the podcast, which limits our ability to ascertain generalizability and reach. Usability and relevance of the model for disadvantaged and high-risk populations also require further investigation.

### Strengths of the Study

A strength of this study was the multilevel qualitative analysis of open-source data, which offered insights into understanding the key characteristics of a successful model for translating evidence into actionable protocols for everyday practice. Mapping qualitative data to the model elements enabled us to confirm the usability and applicability of the model for direct consumers. The confirmatory nature of our analysis also led us to have greater confidence in our results.

Given the user feedback and relative impact of the Active Model of Research Translation, opportunities exist for patient health education and promotion that extend beyond consumer use on the internet. Strategies such as this may have broader implications for engaging patients in their treatment. Examples might include clinicians creating active online communities or health care organizations building dissemination models that are both iterative and interactive for patients and families. Applying this framework and evaluating its impact may help dissemination of evidence-based strategies and targeted interventions across different communities and conditions.

### Conclusions

The HLP offers an innovative way to synthesize scientific evidence and deliver it directly to the general public in the form of actionable tools and an improved understanding of underlying mechanisms. Similar active models of research translation can have significant implications for accessing health information and implementing specific health strategies for improved outcomes. As highlighted elsewhere, the value of active models of research translation has applicability in places and contexts where access to medical or wellness professionals is limited [29].

A lack of practical guidance on the steps required to achieve evidence translation and implementation has impeded many efforts [12]. Accepting 17 years as the average time for research to get translated into practical applications [30] is not compatible with Science Translation 2.0. Developing and utilizing models of research translation that help and empower users should become a standard and stimulate new perspectives for research and policy.

## Abbreviations

HLP: Huberman Lab Podcast
PHR 2.0: Personal Health Records integrated with social networking

## References

[1] Eysenbach G (2008). Google Health starts pilot test at Cleveland Clinic - and my reflections on Personal Health Records 2.0 (PHR 2.0). Gunther Eysenbach Random Rants Blog.

[2] Eysenbach G (2008). Medicine 2.0: social networking, collaboration, participation, apomediation, and openness. J Med Internet Res.

[3] Wong DK, Cheung M (2019). Online health information seeking and eHealth literacy among patients attending a primary care clinic in Hong Kong: a cross-sectional survey. J Med Internet Res.

[4] Fox S, Duggan M (2013). Majority of adults look online for health information. Pew Research Center.

[5] Finney Rutten LJ, Blake KD, Greenberg-Worisek AJ, Allen SV, Moser RP, Hesse BW (2019). Online health information seeking among US adults: measuring progress toward a healthy people 2020 objective. Public Health Rep.

[6] Kyriacou A, Sherratt C (2019). Online health information-seeking behavior by endocrinology patients. Hormones (Athens).

[7] Interactive patient education. Elsevier.

[8] Empowerment for patients. Patient Empowerment Network.

[9] Naslund JA, Aschbrenner KA, Marsch LA, Bartels SJ (2016). The future of mental health care: peer-to-peer support and social media. Epidemiol Psychiatr Sci.

[10] Song S, Zhang Y, Yu B (2021). Interventions to support consumer evaluation of online health information credibility: a scoping review. Int J Med Inform.

[11] Thigpen S, Puddy RW, Singer HH, Hall DM (2012). Moving knowledge into action: developing the rapid synthesis and translation process within the interactive systems framework. Am J Community Psychol.

[12] Hawkins N, Bhuiya A, Shantharam S, Chapel J, Taylor L, Thigpen S (2021). A replicable approach to promoting best practices: translating cardiovascular disease prevention research. J Public Health Manag Pract.

[13] Spencer LM, Schooley MW, Anderson LA, Kochtitzky CS, DeGroff AS, Devlin HM, Mercer SL (2013). Seeking best practices: a conceptual framework for planning and improving evidence-based practices. Prev Chronic Dis.

[14] Ross-Hellauer T, Tennant JP, Banelytė V, Gorogh E, Luzi D, Kraker P, Pisacane L, Ruggieri R, Sifacaki E, Vignoli M (2020). Ten simple rules for innovative dissemination of research. PLoS Comput Biol.

[15] Fontaine G, Maheu-Cadotte M, Lavallée Andréane, Mailhot T, Rouleau G, Bouix-Picasso J, Bourbonnais A (2019). Communicating science in the digital and social media ecosystem: scoping review and typology of strategies used by health scientists. JMIR Public Health Surveill.

[16] Social Blade.

[17] UC2D2CMWXMOVWx7giW1n3LIg's Most Recent YouTube Videos. Social Blade.

[18] Andrew Huberman. YouTube.

[19] Welcome to the Huberman Lab Podcast. YouTube.

[20] Huberman Lab. Huberman Lab/2023 Scicomm Media LLC.

[21] Hsieh H, Shannon SE (2005). Three approaches to qualitative content analysis. Qual Health Res.

[22] Pechmann C (2001). A comparison of health communication models: risk learning versus stereotype priming. Media Psychology.

[23] Glanz K, Rimer BK, Viswanath K (1991). Health Behavior and Health Education: Theory, Research, and Practice (4th ed.).

[24] Greenhalgh T, Wieringa S (2011). Is it time to drop the 'knowledge translation' metaphor? A critical literature review. J R Soc Med.

[25] Chan TM, Dzara K, Dimeo SP, Bhalerao A, Maggio LA (2020). Social media in knowledge translation and education for physicians and trainees: a scoping review. Perspect Med Educ.

[26] Dal Mas F, Garcia-Perez A, Sousa MJ, da Costa RL, Cobianchi L (2020). Knowledge translation in the healthcare sector. A structured literature review. EJKM.

[27] Dal Mas F, Biancuzzi H, Massaro M, Miceli L (2020). Adopting a knowledge translation approach in healthcare co-production. A case study. MD.

[28] Banner D, Bains M, Carroll S, Kandola DK, Rolfe DE, Wong C, Graham ID (2019). Patient and public engagement in integrated knowledge translation research: are we there yet?. Res Involv Engagem.

[29] Lee HY, Jin SW, Henning-Smith C, Lee J, Lee J (2021). Role of health literacy in health-related information-seeking behavior online: cross-sectional study. J Med Internet Res.

[30] Morris ZS, Wooding S, Grant J (2011). The answer is 17 years, what is the question: understanding time lags in translational research. J R Soc Med.

